# The emergence of electron spin in interdisciplinary research in chemistry

**DOI:** 10.1093/nsr/nwae344

**Published:** 2024-10-11

**Authors:** Shang-Da Jiang, Li-Zhu Wu, Song Gao

**Affiliations:** Spin-X Institute, School of Chemistry and Chemical Engineering, State Key Laboratory of Luminescent Materials and Devices, Guangdong-Hong Kong-Macao Joint Laboratory of Optoelectronic and Magnetic Functional Materials, South China University of Technology, China; Key Laboratory of Photochemical Conversion and Optoelectronic Materials, Technical Institute of Physics and Chemistry, Chinese Academy of Sciences, China; Spin-X Institute, School of Chemistry and Chemical Engineering, State Key Laboratory of Luminescent Materials and Devices, Guangdong-Hong Kong-Macao Joint Laboratory of Optoelectronic and Magnetic Functional Materials, South China University of Technology, China; Key Laboratory of Bioinorganic and Synthetic Chemistry of Ministry of Education, Guangdong Basic Research Center of Excellence for Functional Molecular Engineering, School of Chemistry, IGCME, Sun Yat-Sen University, China; Beijing National Laboratory of Molecular Science, Beijing Key Laboratory of Magnetoelectric Materials and Devices, College of Chemistry and Molecular Engineering, Peking University, China

Spin chemistry, by definition, deals with the effects of electron and nuclear spins in the chemical context, and phenomenologically includes all kinds of observations regarding rates and yields of chemical reactions and properties of molecules that are related to the magnetic field. The development of spin chemistry calls for joint efforts involving research communities spanning physics, chemistry, biology, materials science and information science, and will play a crucial role in scientific and technologic innovations. Electron spins in paramagnetic molecules have provided a unique degree of freedom in the study of the physical and chemical properties of molecules. Being a cutting-edge field with challenges implied by its interdisciplinary nature, spin chemistry is worth more attention in the present fundamental and applied fundamental studies.

The Special Topic of ‘spin chemistry’ is to introduce and discuss the frontiers of spin chemistry, emphasizing the recent developments in this important research field. We have collected 11 papers in this Special Topic, including 1 Research Highlight, 3 Perspectives, 2 Research Articles, 4 Reviews and 1 Interview. For the Research Highlight, Sessler [1] introduces the magnetic field effects in living cells, studied by translating knowledge obtained from *in vitro* studies of radical pair chemistry, as reported in the Research Article by Zhang and Gao [2], which is also included in this Special Topic. The other Research Article, by Cui *et al*., represents the pioneering utilization of covalent organic frameworks as spin-dependent catalysts for electrochemical oxygen evolution reaction, showcasing the immense potential of covalent organic frameworks for electron spin polarization and their application in catalysis [3].

For the 3 Perspectives, Yu and Tian overview recent research progress and prospects in elucidating the catalytic mechanism of photoenzymes by using electron spin resonance spectroscopy, which is emerging as a unique and crucial method for identifying radical intermediates, illustrating electron-transfer events and the underlying mechanisms of photoenzymatic catalysis [4]. Hore focuses on the power of spin chemistry beyond chemical systems. Already having provided insights into the structures and dynamics of molecules and the kinetics and mechanisms of their reactions, spin chemistry is believed to achieve more goals in living systems, in areas such as the physiological effects of anthropogenic magnetic fields, the effectiveness of magnetic therapies and medical diagnostics, and the improvement of crop yields [5]. Lu *et al*. explores detailed structural design and strategies for spin regulation in single-atom spin catalysis, enabling unparalleled efficiency in chemical transformations through the harnessing of spin effects combined with atomic precision of active sites [6].

For the 4 Reviews, Waldeck, Lu and co-workers discuss the opportunities in chemistry that are enabled by the chiral induced spin selectivity effect, which provides a new strategy for spin control in enantioselective chemical reactions and separations [7]. Wu, Zhang and co-workers provide an overview of the concept of spin photochemistry, and summarize some recent advances on spin-related excited state phenomena in terms of magnetism, luminescence and reactivity [8]. Zhou, Liu and co-workers summarize the recent research progress in detecting biomarkers in the tumor microenvironment by using dynamic nuclear polarization techniques and discuss future directions of development in this field [9]. Xu *et al*. present the current understanding of spin-dependent electrocatalysis, highlighting key aspects such as spin features in electrocatalysts, techniques for spin characterization, strategies for spin manipulation, as well as the challenges and future perspectives in this emerging field [10].

Finally, one of the guest editors of this Special Topic, Prof. Song Gao, was interviewed by *National Science Review* [11]. Gao discussed the significance of electron spins in chemistry research, spin research characteristics from a molecular point of view and the most important scientific questions in spin chemistry. We hope that this Special Topic about spin chemistry will inspire further achievements in this field and, moreover, will encourage more scientists to devote more efforts to spin chemistry, and thereby develop further progress in spin chemistry research.

***Conflict of interest statement.*** None declared.
